# Fractionation of Acacia Wood and Sugarcane Bagasse by Levulinic Acid–Choline Chloride Deep Eutectic Solvent Treatment Followed by Alkaline Washing

**DOI:** 10.3390/polym18212729

**Published:** 2026-11-03

**Authors:** Solange Magalhães, María José Aliaño-González, Sofia Leão, Margarida Oliveira, Inês Neves, Claudio Lima, Bruno Medronho, Luís Alves, Maria da Graça Rasteiro

**Affiliations:** 1 Surface and Colloid Engineering, FSCN Research Centre, Mid Sweden University, SE-851 70 Sundsvall, Sweden; bfmedronho@ualg.pt; 2 Department of Chemical Engineering, Chemical Engineering and Renewable Resources for Sustainability, CERES, University of Coimbra, Rua Sílvio Lima, Pólo II, Pinhal de Marrocos, 3030-790 Coimbra, Portugal; sofialeao4@gmail.com (S.L.); moliveira@uc.pt (M.O.); ines.neves@uc.pt (I.N.); luisalves@ci.uc.pt (L.A.); mgr@eq.uc.pt (M.d.G.R.); 3 Department of Analytical Chemistry, Faculty of Sciences, University of Cadiz, Agrifood Campus of International Excellence (ceiA3), IVAGRO, 11510 Puerto Real, Spain; 4 GS4Science Co., Rua Barão de Piracicamirim, 1645, Piracicaba 13146-389, SP, Brazil; claudio.gs4science@gmail.com; 5 MED—Mediterranean Institute for Agriculture, Environment and Development, CHANGE—Global Change and Sustainability Institute, Faculdade de Ciências e Tecnologia, Universidade do Algarve, Campus de Gambelas, Ed. 8, 8005-139 Faro, Portugal

**Keywords:** biomass valorization, deep eutectic solvents, lignin extraction, acacia wood, sugarcane bagasse, multiresponse optimization

## Abstract

Deep eutectic solvents (DESs) are versatile media for lignocellulosic biomass fractionation, but their practical relevance depends on whether they remain effective across feedstocks of very different structure and composition. In this study, DESs composed of levulinic acid and choline chloride (LA:ChCl) at molar ratios of 2:1 and 3:1 were applied to two structurally distinct lignocellulosic sources, Acacia wood and sugarcane bagasse, to assess the applicability of the same solvent system to feedstocks of contrasting structure rather than its performance on a single substrate. Extractions were performed at 120, 140, and 160 °C for 1, 2, 4, 6, and 8 h, and process performance was assessed through recovered solid yield and total lignin content in the recovered solid fraction obtained after DES treatment followed by alkaline washing. The combined DES treatment and subsequent alkaline washing resulted in substantial lignin removal from both feedstocks, although the individual contribution of each treatment step could not be determined. For Acacia wood, recovered solid yields of up to 89.20 ± 0.07% and lignin contents as low as 0.94 ± 0.21% were achieved, while for sugarcane bagasse a 67.48 ± 0.15% recovered solid yield and 1.32 ± 0.77% total lignin content were obtained at a 3:1 ratio, 140 °C, and 6 h. A four-factor multivariate analysis revealed a significant Feedstock × Ratio × Time × Temperature interaction, indicating that, although the solvent was effective for both types of biomass, the operational window that maximizes its performance must be tuned to the specific feedstock: Acacia wood exhibited a pronounced trade-off between solid recovery and lignin removal, whereas sugarcane bagasse allowed relatively high recovered solid yields and low lignin contents to be achieved simultaneously. Overall, these results demonstrate the applicability of the LA:ChCl-based fractionation process, including subsequent alkaline washing, to the two structurally distinct feedstocks investigated, while highlighting the need to determine the individual contribution of each treatment step.

## 1. Introduction

The growing demand for sustainable alternatives to fossil-based resources has intensified interest in lignocellulosic biomass as a renewable feedstock for the production of chemicals, fuels, polymers, and functional materials [1,2]. As the most abundant source of renewable carbon on Earth, lignocellulosic biomass offers considerable potential for the development of integrated biorefinery strategies in which its major components—cellulose, hemicelluloses, and lignin—are selectively separated and converted into value-added products [3]. In particular, recovered solid fractions are attractive for the production of fibers, films, composites, and advanced materials, while lignin has gained increasing relevance as a renewable aromatic resource for resins, adhesives, carbon materials, and polymer formulations [2,4]. However, the efficient use of lignocellulosic biomass remains challenging because of the complex and recalcitrant architecture of plant cell walls [5].

This recalcitrance arises from the intimate association between cellulose microfibrils, amorphous hemicelluloses, and the highly crosslinked lignin network [6,7]. The structural role of lignin, together with the extensive hydrogen-bonding network within cellulose and the heterogeneous arrangement of cell wall polymers, considerably hinders solvent penetration and limits the selective recovery of biomass fractions [8,9]. As a result, effective fractionation is a crucial step in lignocellulosic biomass valorization, since it determines not only the separation efficiency of lignin and carbohydrate-rich fractions but also the physicochemical properties and potential end uses of the recovered components.

Conventional fractionation processes, including kraft pulping, sulfite pulping, and organosolv treatments, have demonstrated the feasibility of biomass deconstruction at industrial scale [10]. Nevertheless, these approaches often involve harsh operating conditions, corrosive chemicals, high energy demands, or solvent systems with environmental and economic limitations [11]. In recent years, alternative solvent-based strategies have therefore been explored to improve the sustainability and selectivity of biomass pretreatment and fractionation. Among these, deep eutectic solvents (DESs) have emerged as promising media due to their simple preparation, low vapor pressure, tunable properties, and potential compatibility with green chemistry principles [12].

DESs are generally formed by combining a hydrogen bond acceptor and a hydrogen bond donor, resulting in a eutectic mixture with a melting point significantly lower than that of the individual components [13]. Their physicochemical behavior can be tailored by selecting suitable components and molar ratios, enabling the design of solvent systems with specific acidity, polarity, viscosity, and hydrogen-bonding capacity [14]. In the context of lignocellulosic biomass processing, DESs have attracted significant attention because they can disrupt lignin-carbohydrate interactions, promote lignin solubilization, and enrich the cellulose content in the residual solid fraction [15]. Depending on their composition, DESs may also preserve or modify structural features of the recovered lignin and cellulose, which is particularly relevant for subsequent applications in materials and polymeric systems [16]. Among the different DES formulations reported for biomass processing, acidic DESs have shown particular effectiveness for delignification [17]. Their ability to promote cleavage of ether linkages, weaken supramolecular interactions in the cell wall, and facilitate lignin extraction makes them highly attractive as fractionation media [18]. In this regard, levulinic acid (LA) is a particularly interesting hydrogen bond donor because it is a biomass-derived platform molecule, reinforcing the renewable character of the solvent system [19,20]. When combined with choline chloride (ChCl), a low-cost and widely used hydrogen bond acceptor, it forms a DES with promising potential for sustainable biomass fractionation [21]. Beyond extraction efficiency, such systems are especially relevant when they enable the recovery of lignin-rich and cellulose-enriched fractions that can be directly considered for value-added applications.

Despite the increasing number of studies on DES-based fractionation, many investigations are still limited to a single biomass source. This restricts the broader assessment of solvent robustness, since lignocellulosic feedstocks differ substantially in chemical composition and ultrastructural organization. Hardwoods, softwoods, and agricultural residues present distinct lignin contents, syringyl/guaiacyl distributions, hemicellulose compositions, ash contents, porosity, and cell wall arrangements, all of which may strongly influence solvent accessibility, delignification kinetics, and fraction recovery. Consequently, a DES that performs well for one biomass type may not necessarily show the same efficiency for another. Demonstrating comparable fractionation performance across structurally distinct feedstocks is therefore essential to support the practical applicability of DES systems in flexible and scalable biorefinery schemes.

From this perspective, evaluating feedstock dependence is not only a matter of process performance but also of technological relevance. A fractionation method that operates efficiently under the same conditions for different biomass sources offers clear advantages in terms of operational simplicity, process transferability, and raw-material flexibility. Such robustness is particularly important for future industrial implementation, where seasonal availability, regional diversity, and supply-chain variability often require the use of multiple lignocellulosic resources. A solvent system with low sensitivity to biomass source would therefore represent a significant step toward more adaptable and economically viable biomass processing platforms.

In this work, DES composed of LA and ChCl at molar ratios of 2:1 and 3:1 were evaluated on two structurally distinct lignocellulosic feedstocks: Acacia wood residues and sugarcane bagasse, to evaluate whether the same solvent system is effective for both substrates. The influence of extraction temperature (120, 140, and 160 °C) and treatment time (1, 2, 4, 6, and 8 h) was investigated using recovered solid yield and total lignin content in the recovered solid fraction as response variables.

This work builds on our previous studies, in which LA:ChCl was applied to Acacia wood using a reduced Box–Behnken design to optimize cellulose and lignin yields and to evaluate solvent reuse [21], and to characterize the resulting fractions [19], and in which a single LA:ChCl condition was used to isolate cellulose from sugarcane bagasse [22]. In contrast to those studies, which addressed a single feedstock or a single operating condition, the present work applies a full factorial design (2 molar ratios × 5 extraction times × 3 temperatures) to both feedstocks under identical experimental and analytical procedures. This makes it possible to test statistically whether, and how, the effects of solvent composition, temperature, and time depend on the feedstock (i.e., the Feedstock × Ratio × Time × Temperature interaction), and to compare the shape of the trade-off between solid recovery and delignification for each feedstock through Pareto and desirability analyses. Such a direct, statistically supported assessment of the feedstock dependence of an LA:ChCl process provides a quantitative basis for deciding whether a single DES can be operated in multi-feedstock biorefinery schemes and how its operating window must be adapted to each feedstock.

The main objective was to evaluate whether the LA:ChCl solvent system is effective for lignin extraction from two feedstocks of contrasting structure and composition, while also determining how solvent composition, temperature, and extraction time should be tuned to maximize fractionation performance for each feedstock. Particular attention was given to the trade-off between solid recovery and lignin removal through factorial statistical analysis, Pareto optimization, and an equal-weight desirability approach.

## 2. Materials and Methods

### 2.1. Materials

Sugarcane bagasse (*Saccharum officinarum*) was supplied by the Hugot Laboratory of Sugar Technology, University of São Paulo, Piracicaba, Brazil. Its average chemical composition, determined as described in Section 2.3, consisted of 38.78% cellulose, 23.81% hemicellulose, and 24.02% lignin. Prior to fractionation, the material was milled using a knife mill (Thomas Scientific, Swedesboro, NJ, USA) to an average particle size of 0.84 mm. Acacia wood (*Acacia dealbata*) was collected in Midões (Tábua), Portugal. The raw material, consisting of waste branches, was milled in the same knife mill and size-classified using a mechanical sieve shaker (Thomas Scientific, Swedesboro, NJ, USA). The fraction with particle size between 0.25 and 0.84 mm was selected for subsequent pretreatment. The chemical composition of both raw materials, determined following the same procedure [19,22], is shown in Table 1. The DES components, LA (98 wt%) and ChCl (99 wt%), were purchased from Acros Organics (Geel, Belgium). Sulfuric acid (72 wt%) was obtained from Chem-Lab NV (Zedelgem, Belgium). A 10 wt% NaOH solution (LabKem Premià de Dalt, Spain) was prepared with distilled water.

### 2.2. DES Fractionation Procedure

Fractionation of Acacia wood and sugarcane bagasse was performed using DESs composed of LA and ChCl at molar ratios of 2:1 and 3:1. For each experiment, 0.75 g of oven-dried biomass was mixed with 10 mL of DES in a cylindrical metallic reactor suitable for high-temperature operation. Extractions were conducted at 120, 140, or 160 °C for 1, 2, 4, 6, or 8 h. Two independent extractions were carried out for each combination of feedstock, DES ratio, extraction time, and temperature. At the end of the treatment, the reactor was cooled to room temperature and the solid and liquid phases were separated. The resulting solid fraction was washed with distilled water to promote the transfer of solubilized lignin into the liquid phase, and both fractions were recovered by vacuum filtration. The solid fraction was subsequently washed with a 10 wt% NaOH aqueous solution, to remove residual lignin associated with the fibres, and then repeatedly washed with distilled water until neutral pH was achieved. Typically, 5–6 washing cycles were required. The alkaline washing was performed at room temperature. This step is an integral part of the fractionation sequence and may itself contribute to lignin removal; therefore, the recovered solid yields and total lignin contents reported in this work reflect the combined effect of the DES treatment and the subsequent alkaline washing, and not of the DES treatment alone. Both the recovered solid and lignin-rich fractions were finally dried at 50–60 °C for 24 h before compositional analysis.

### 2.3. Chemical Composition of Raw Materials and Determination of Total Lignin Content

The lignin content of the raw materials and recovered solid fractions was determined according to the NREL laboratory analytical procedure NREL/TP-510-42618 [23] (version 08-03-2012). Approximately 300 mg of sample was hydrolysed with 3 mL of 72 wt% H_2_SO_4_ at 30 °C for 1 h under stirring. The mixture was subsequently diluted to an acid concentration of 4 wt% and autoclaved at 121 °C for 1 h. After hydrolysis, the suspension was vacuum-filtered through preweighed crucibles, and acid-insoluble lignin was determined gravimetrically. Acid-soluble lignin was quantified by UV–Vis spectrophotometry at 205 nm using an absorptivity of 110 L g^−1^ cm^−1^. Total lignin content was calculated as the sum of acid-insoluble and acid-soluble lignin expressed as a percentage of the dry raw material or of the recovered solid fraction, as applicable.

For the raw materials, structural carbohydrates were additionally quantified in the hydrolysates obtained in the same two-step acid hydrolysis, following the NREL procedure [23], as previously reported for these feedstocks [19,22]. Monosaccharides were analysed by high-performance liquid chromatography (HPLC) with a refractive index detector. For Acacia wood, a Rezex ROA-Organic Acid H+ column (Phenomenex, Torrance, CA, USA) was used with 0.0025 M H_2_SO_4_ as the mobile phase [19]. For sugarcane bagasse, the hydrolysates were neutralised with CaCO_3_ to pH 5–6, filtered through 0.22 µm membranes, and analysed on an Agilent Hi-Plex Ca column (300 × 7.7 mm; Agilent Technologies, Santa Clara, CA, USA) at 0.6 mL min^−1^, using ultrapure water as the mobile phase [22]. Cellulose and hemicellulose contents were calculated from the glucose and xylose concentrations, respectively, and all compositional data were expressed on a dry-biomass basis.

### 2.4. Determination of Recovered Solid Yield

The yield of the recovered solid fraction was calculated after completion of the fractionation and washing procedures. As mentioned, the recovered solid was dried at 50–60 °C for 24 h and weighed after cooling to room temperature. The yield was expressed relative to the initial dry mass of biomass according to Equation (1):

(1) $$Recovered\;solid\;yield\;\left(\%\right)=\;\frac{m_{recovered\;solid}}{m_{initial\;dry\;biomass}}\times 100$$

where *m*_recovered solid_ is the dry mass of the solid fraction obtained after DES treatment, filtration, water washing, alkaline washing, and final drying, and *m*_initial dry biomass_ is the initial oven-dried biomass mass used in the experiment. As mentioned, two independent extractions were performed for each combination of feedstock, LA:ChCl molar ratio, extraction time, and temperature, and the results were expressed as mean ± standard deviation. It should be noted that the recovered solid yield integrates the removal of lignin, the dissolution or degradation of carbohydrates, the removal of extractives and ash, and any material losses during the washing steps; therefore, it describes the overall solid recovery and should not be interpreted as cellulose recovery.

### 2.5. Data Analysis

Statistical analyses were performed using R software, version 4.6.1. The experimental design included four factors: feedstock type (Acacia wood or sugarcane bagasse), LA:ChCl molar ratio (2:1 or 3:1), extraction time (1, 2, 4, 6, or 8 h), and extraction temperature (120, 140, or 160 °C). Two independent extractions were analysed for each combination of experimental conditions.

The response variables considered were the recovered solid yield and total lignin content in the recovered solid fraction. Total lignin was calculated for each independent extraction as the sum of the acid-insoluble and acid-soluble lignin fractions. The limits of detection (LOD) and quantification (LOQ) of the lignin determination were 0.14% and 0.47% respectively. Values below the LOD, which occurred in 2 of the 120 individual determinations (all corresponding to (acid-soluble/acid-insoluble) lignin under (conditions)), were assigned a value of zero for the calculation of total lignin. Because this substitution may bias means, variances, and factorial effects, a sensitivity analysis was performed in which these values were replaced by LOD/2 and by the LOD.

An initial four-factor multivariate analysis of variance (MANOVA) was performed using recovered solid yield and total lignin as joint response variables. Pillai’s trace was selected as the multivariate test statistic. Subsequently, separate four-factor linear models were fitted for each response according to: Feedstock × Ratio × Time × Temperature.

Because significant interactions involving feedstock type were detected, additional three-factor models were fitted separately for Acacia wood and sugarcane bagasse: Ratio × Time × Temperature.

Time and temperature were treated as categorical factors in the factorial analyses to avoid assuming a linear or quadratic relationship between the operating conditions and the responses. Type III sums of squares were used because the interpretation focused on interactions between experimental factors. Classical ANOVA results were complemented with heteroscedasticity-consistent HC3 tests. The robust results were used to confirm the statistical conclusions because deviations from residual normality and differences in residual dispersion were observed in some models, particularly regarding the lignin content from sugarcane bagasse. It should also be noted that only two independent extractions were performed for each condition. Although the full factorial structure provides 60 residual degrees of freedom for the four-factor models (30 for each feedstock-specific model), the variability within each condition is estimated from only two observations, which limits the power to detect small differences and reduces the precision of heteroscedasticity-consistent estimators. For this reason, the interpretation focused on effects that were significant in both the classical and the HC3-robust analyses, effect sizes were reported alongside *p*-values, and condition-specific pairwise comparisons were interpreted with caution, particularly for small differences.

When significant interactions were identified, simple-effects comparisons were performed using estimated marginal means. The two solvent ratios were compared within each combination of feedstock, extraction time, and temperature using Holm-adjusted *p*-values. Comparisons among extraction times and temperatures were adjusted using Tukey’s method. Acacia wood and sugarcane bagasse were compared only under identical ratio, time, and temperature conditions. Partial eta-squared values were calculated from the classical models as measures of effect size.

Multiresponse optimization was evaluated using Pareto analysis, with recovered solid yield as the response to be maximized and total lignin content in the recovered solid fraction as the response to be minimized. A condition was considered Pareto-optimal when no other experimental condition simultaneously provided a higher recovered solid yield and a lower total lignin content. An overall desirability function was also calculated using equal weights for recovered solid yield and lignin content. For each feedstock, individual desirabilities were obtained by linear scaling of each response between its minimum and maximum mean values observed in the experimental domain: for recovered solid yield (Y, maximized), dY = (Y − Ymin)/(Ymax − Ymin); for total lignin content (L, minimized), dL = (Lmax − L)/(Lmax − Lmin). The overall desirability was calculated as the geometric mean of the individual desirabilities, D = (dY × dL)1/2, which corresponds to assigning equal weights to both responses. Time and temperature were not penalized in the primary desirability calculation; consequently, the maximum desirability identifies the best experimental compromise between the two responses, and not necessarily an energetic or economic process optimum. Quadratic response-surface models were evaluated through lack-of-fit tests but were not used for continuous optimization when significant lack of fit was detected. Statistical significance was established at *p* < 0.05.

Both Pareto and desirability analyses were based on the mean responses obtained from two independent extractions per experimental condition. Since no additional confirmation experiments were performed, the selected conditions represent the best-performing combinations within the investigated experimental domain rather than independently validated process optima.

## 3. Results and Discussion

### 3.1. Overall Influence of Feedstock and Operating Conditions

To obtain an initial integrated overview of the fractionation process, recovered solid yield and total lignin content in the recovered solid fraction were first analysed simultaneously by means of a four-factor MANOVA. This approach was selected because both responses describe complementary aspects of fractionation performance: recovered solid yield reflects the amount of recovered solid material, whereas total lignin content characterizes the lignin remaining in the recovered solid after the complete fractionation and washing procedure.

The MANOVA showed that the combined response was significantly affected by the interaction between feedstock, LA:ChCl ratio, extraction time, and temperature (Pillai’s trace = 0.985, F(16,120) = 7.28, *p* = 1.48 × 10^−11^). This result indicates that the two biomasses did not respond uniformly to changes in the operating conditions. In other words, the influence of the solvent ratio, extraction time, and temperature depended on whether Acacia wood or sugarcane bagasse was treated.

Following this global analysis, recovered solid yield and total lignin content were examined separately using four-factor factorial models. The Feedstock × Ratio × Time × Temperature interaction remained significant in the HC3-robust analyses for both recovered solid yield (F(8,60) = 5.38, *p* = 4.08 × 10^−5^) and total lignin content (F(8,60) = 3.38, *p* = 0.0029). The corresponding partial eta-squared values obtained from the classical models were 0.579 and 0.384, respectively, indicating that this interaction accounted for a substantial proportion of the variation observed in both responses.

These findings show that the main effects of solvent ratio, extraction time, and temperature cannot be interpreted independently of one another. For example, a ratio that improves delignification at a particular temperature may not produce the same effect at another treatment time or when applied to a different biomass. For this reason, subsequent analyses were performed separately for Acacia wood and sugarcane bagasse.

**Figure 1:**
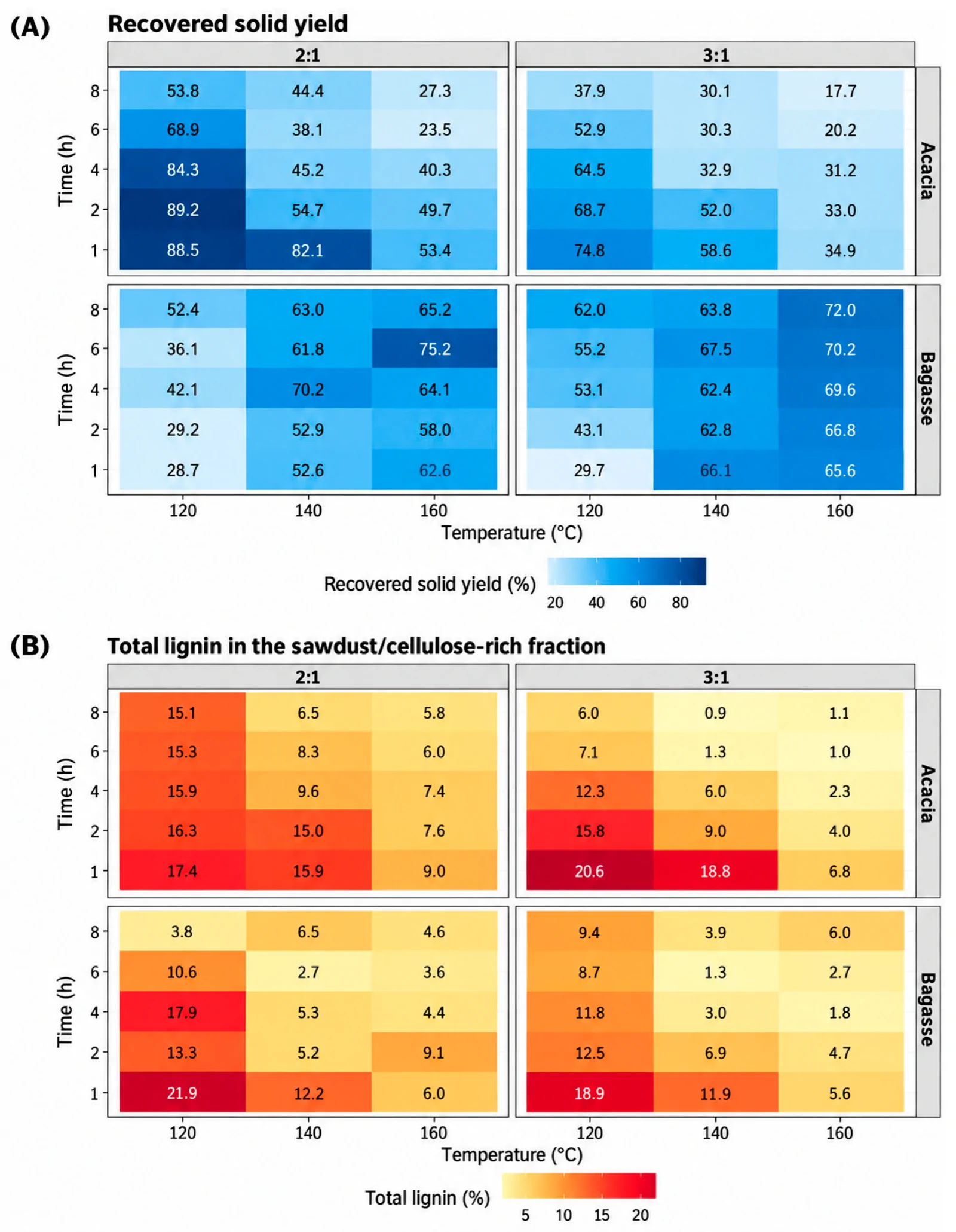
Heat maps showing (**A**) mean recovered solid yield and (**B**) mean total lignin content in the recovered solid fraction obtained from Acacia wood and sugarcane bagasse using LA:ChCl molar ratios of 2:1 and 3:1. Treatments were performed for 1, 2, 4, 6, or 8 h at 120, 140, or 160 °C. Values represent the means of two independent extractions.

The experimental response maps for recovered solid yield and total lignin content in the recovered solid fraction are presented in Figure 1, while the complete numerical results for all experimental conditions are provided in Table S1.

### 3.2. Fractionation Behaviour of Acacia Wood

Once the feedstocks were analysed independently, Acacia wood showed a highly condition-dependent fractionation behaviour. The interaction between LA:ChCl ratio, time, and temperature was significant for both recovered solid yield (HC3: F(8,30) = 6.13, *p* = 0.00011) and total lignin content in the recovered solid fraction (HC3: F(8,30) = 20.62, *p* = 3.29 × 10^−10^). Therefore, the effect of changing the solvent ratio varied according to the severity of the treatment.

When the results were averaged descriptively across all extraction times and temperatures, the 2:1 ratio produced a higher mean recovered solid yield than the 3:1 ratio, with values of 56.23% and 42.64%, respectively. However, the opposite trend was observed for total lignin content in the recovered solid fraction. The mean lignin content was 11.41% for the 2:1 ratio and 7.54% for the 3:1 ratio. These results suggest that increasing the proportion of LA was associated with lower residual lignin contents after the complete fractionation and washing procedure. Nevertheless, because of the significant three-factor interaction, the individual experimental conditions provide a more informative interpretation than the overall means.

The highest recovered solid yield for Acacia wood was obtained using the 2:1 ratio at 120 °C for 2 h, reaching 89.20 ± 0.07%. Despite the high solid recovery, the resulting fraction still contained 16.26 ± 0.20% total lignin. Therefore, this condition favoured the preservation of the solid fraction while not providing extensive delignification.

At the opposite end of the experimental domain, the lowest lignin content was obtained using the 3:1 ratio at 140 °C for 8 h. Under these conditions, the total lignin content in the recovered solid fraction decreased to 0.94 ± 0.21%. However, the recovered solid yield was only 30.05 ± 1.65%, indicating that the extensive removal of lignin was accompanied by a considerable reduction in recovered solid mass.

The existence of this compromise can also be observed under intermediate conditions. At 120 °C for 6 h, the 2:1 ratio produced a recovered solid yield of 68.86 ± 0.13% and a total lignin content of 15.30 ± 0.21%. When the solvent ratio was changed to 3:1, the recovered solid yield decreased to 52.93 ± 1.70%, whereas the total lignin content in the recovered solid fraction was reduced to 7.14 ± 0.36%. Both differences were statistically significant after Holm correction (*p* < 0.001). Thus, under these conditions, the 3:1 ratio removed approximately half of the lignin remaining with the 2:1 treatment, but this improvement was achieved at the expense of approximately 16 percentage points of recovered solid yield.

Taken together, these results demonstrate that the fractionation of Acacia wood implies a pronounced trade-off between solid recovery and delignification. Relatively mild conditions and the 2:1 ratio generally favoured higher recovered solid yields, whereas treatments involving the 3:1 ratio and increased harshness promoted lower total lignin content in the recovered solid fraction.

**Figure 2:**
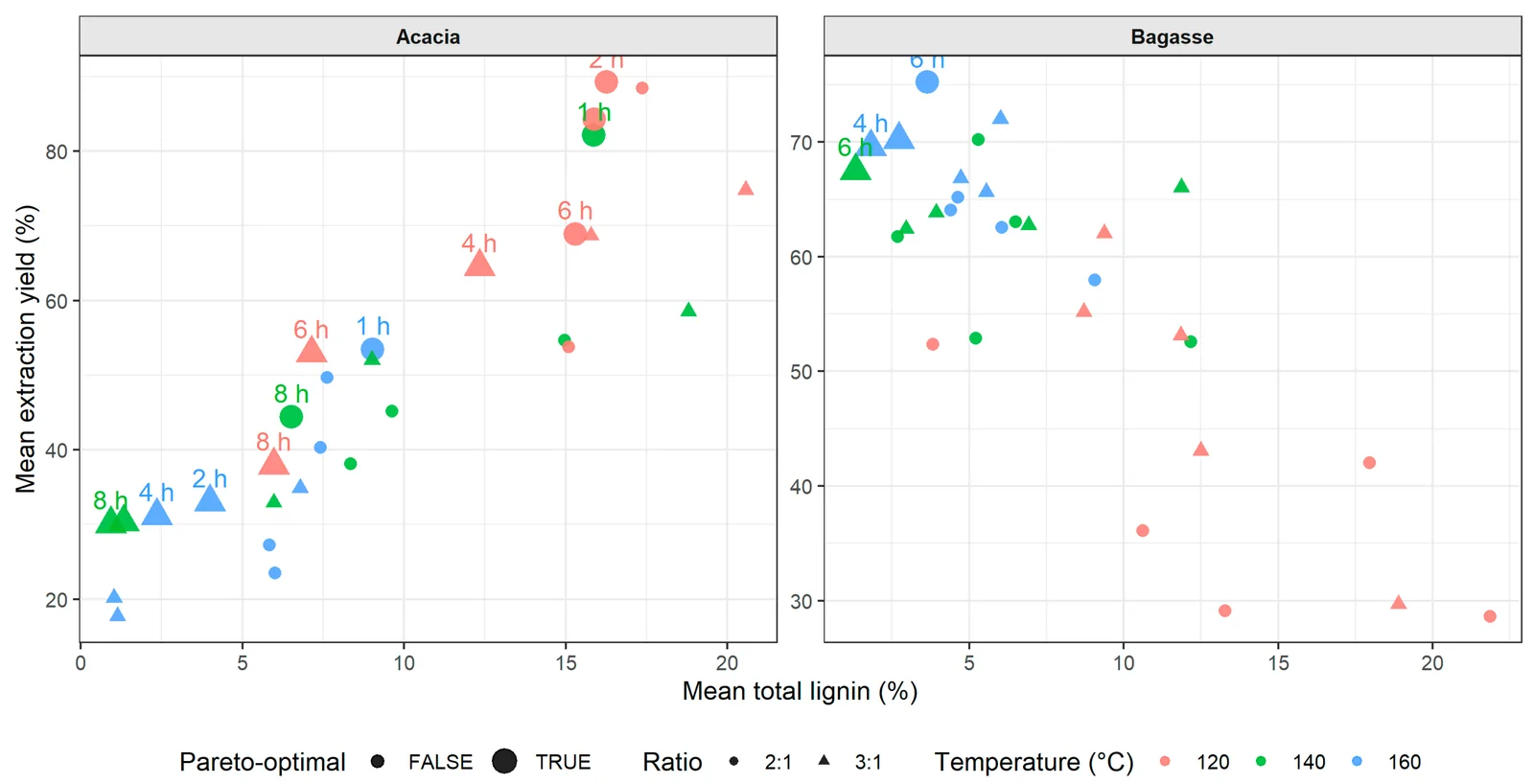
Multiresponse optimization of the fractionation of Acacia wood and sugarcane bagasse using LA:ChCl deep eutectic solvents. Each point represents the mean recovered solid yield and total lignin content in the recovered solid fraction obtained under one experimental condition. Point colour indicates extraction temperature, point shape indicates the LA:ChCl molar ratio, and labels indicate extraction time. Enlarged points represent Pareto-optimal conditions for which recovered solid yield could not be increased without simultaneously increasing total lignin content in the recovered solid fraction. Pareto-optimal conditions were identified among the experimentally evaluated conditions only and do not represent model-predicted optima.

To examine this compromise more systematically, the experimental conditions were subjected to a Pareto analysis in which the recovered solid yield was maximized and the total lignin content in the recovered solid fraction was minimized. A total of 13 Acacia wood conditions were identified as Pareto-optimal (Figure 2). It should be emphasized that the Pareto analysis was performed on the experimentally evaluated conditions only; the Pareto-optimal conditions therefore represent the best compromises among the tested design points and not predicted global process optima, since the response-surface models showed significant lack of fit (Section 3.4). The relatively broad Pareto front confirms that no single treatment simultaneously maximized solid recovery and minimized lignin content. Instead, the most appropriate condition depends on the intended downstream application of the recovered fraction.

When equal importance was assigned to both responses using a desirability function, the highest overall desirability was obtained with the 3:1 ratio at 120 °C for 6 h. This treatment provided a recovered solid yield of 52.93 ± 1.70% and a total lignin content in the recovered solid fraction of 7.14 ± 0.36%. This condition may therefore be considered a balanced operational compromise within the investigated experimental domain, although conditions providing either greater solid recovery or more extensive delignification may be preferable for specific applications.

### 3.3. Fractionation Behaviour of Sugarcane Bagasse

Sugarcane bagasse showed a different fractionation pattern from that observed for Acacia wood. For recovered solid yield, the Ratio × Time × Temperature interaction remained significant in the robust model (F(8,30) = 3.64, *p* = 0.0045). This indicates that the influence of the solvent ratio on the recovery of the solid fraction changed depending on the treatment time and temperature.

In contrast, the three-factor interaction was not significant for total lignin content in the recovered solid fraction (F(8,30) = 1.29, *p* = 0.287). The main effect of the solvent ratio was also not statistically significant after robust correction (F(1,30) = 2.65, *p* = 0.114). Instead, lignin removal from sugarcane bagasse was governed mainly by the extraction time (F(4,30) = 22.63, *p* = 1.07 × 10^−8^), temperature (F(2,30) = 69.38, *p* = 5.59 × 10^−12^), and the interaction between time and temperature (F(8,30) = 10.76, *p* = 5.43 × 10^−7^).

The predominance of temperature, extraction time, and their interaction over the LA:ChCl molar ratio may tentatively be attributed to the hierarchical and supramolecular organization of sugarcane bagasse. The lignocellulosic matrix comprises highly ordered cellulose microfibrils, predominantly in the cellulose I crystalline form, embedded in a hemicellulose-rich matrix and surrounded by an amorphous, three-dimensional lignin network interconnected through hydrogen bonding and lignin–carbohydrate complexes (LCCs) [24,25]. Accordingly, DES-mediated delignification involves solvent penetration and diffusion into the biomass matrix, disruption of hydrogen-bonding interactions and LCC linkages, and subsequent lignin solubilization. Increasing temperature enhances DES molecular mobility, reduces viscosity, improves mass transfer, and accelerates the physicochemical interactions responsible for lignin removal, whereas longer residence times allow these processes to progress toward a greater extent of fractionation [26,27]. Thus, within the experimental range investigated, the LA:ChCl molar ratio was apparently not the limiting factor, while temperature and extraction time predominantly governed the kinetics and extent of delignification. It should be noted, however, that these structural features were not characterized in the present study; this interpretation is therefore a literature-based hypothesis rather than an experimentally demonstrated mechanism.

These results suggest that, unlike Acacia wood, increasing treatment severity was more influential than changing the LA:ChCl ratio for reducing the total lignin content in the recovered solid fraction of the bagasse. Nevertheless, the significant interactions affecting recovered solid yield show that the solvent ratio still influenced the overall fractionation performance under specific combinations of time and temperature.

When averaged across all experimental conditions, the recovered solid yield was 54.27% for the 2:1 ratio and 60.67% for the 3:1 ratio. The corresponding mean total lignin contents were 8.47% and 7.27%, respectively. Although these descriptive averages suggest a generally favourable performance of the 3:1 ratio, the condition-specific comparisons showed that this advantage was not uniform throughout the experimental domain.

Under the mildest treatment conditions, corresponding to the 2:1 ratio at 120 °C for 1 h, the recovered solid yield was only 28.66 ± 0.14%, while the recovered fraction retained 21.86 ± 0.14% total lignin. This result indicates limited biomass deconstruction and delignification. As treatment temperature and time increased, recovered solid yield generally improved and total lignin content in the recovered solid fraction decreased markedly.

The increase in recovered solid yield with treatment severity observed for sugarcane bagasse is counterintuitive, since harsher conditions would be expected to solubilize a larger fraction of the biomass, as observed for Acacia wood. This behaviour was not investigated experimentally in the present study, and the following explanations should therefore be regarded as hypotheses. First, under mild conditions, the DES may swell and partially deconstruct the bagasse without solubilizing a substantial fraction of its hemicelluloses and lignin, which would then be dissolved during the subsequent 10 wt% NaOH washing; grass xylans and lignins are particularly alkali-soluble, and the alkaline step may also release fines from the parenchyma-rich bagasse that are lost during the repeated filtrations. Under this hypothesis, the low yield obtained under mild conditions would be largely determined by the alkaline washing rather than by the DES treatment, which is consistent with the apparent lignin removal of approximately 74% calculated for the mildest condition (2:1, 120 °C, 1 h) despite its low severity. Second, under more severe conditions, the solid may gain mass or become less alkali-soluble through the esterification of carbohydrate and lignin hydroxyl groups by LA, the deposition of condensation products formed from carbohydrates and lignin, or the retention of DES components. Distinguishing between these possibilities requires the determination of the solid yield and composition before and after alkaline washing, the ash and carbohydrate contents of the recovered solids, and spectroscopic evidence of ester formation. Importantly, the unresolved origin of this unexpected behaviour introduces uncertainty into the interpretation of the multiresponse optimization. Since recovered solid yield was determined after the complete fractionation and washing procedure, higher solid recovery under more severe conditions cannot necessarily be interpreted as improved preservation of structural carbohydrates or greater fractionation selectivity. The measured yield may also be influenced by differences in washing and filtration losses, changes in the composition of the recovered material, or the retention of additional compounds. Consequently, the Pareto-optimal and maximum-desirability conditions identified for sugarcane bagasse should be regarded as the most favourable experimental compromises between the two measured responses within the investigated domain, rather than as validated process optima. Further experiments, including the determination of solid recovery before and after alkaline washing, complete component mass balances, and compositional characterization of the recovered fractions, would be required to clarify this behaviour and confirm the reliability of the selected operating conditions.

The highest recovered solid yield was obtained using the 2:1 ratio at 160 °C for 6 h. Under these conditions, the recovered solid yield reached 75.20 ± 3.07%, while the total lignin content in the recovered solid fraction was 3.64 ± 0.65%. This condition also presented the highest overall desirability when recovered solid yield and total lignin content in the recovered solid fraction were assigned equal weights.

The lowest lignin content was observed using the 3:1 ratio at 140 °C for 6 h. This treatment produced a fraction containing only 1.32 ± 0.77% total lignin while maintaining a recovered solid yield of 67.48 ± 0.15%. Compared with the corresponding 2:1 condition, the mean values were more favourable for both responses. However, the differences between the two ratios at 140 °C and 6 h were not statistically significant after Holm correction. Therefore, the apparent advantage of the 3:1 ratio under these conditions should be interpreted as a descriptive tendency rather than a statistically demonstrated difference.

A particularly relevant result was obtained at 160 °C for 4 h. Under these conditions, the 3:1 ratio produced a recovered solid yield of 69.58 ± 0.11% and a total lignin content in the recovered solid fraction of 1.81 ± 0.12%. In comparison with the 2:1 ratio at the same time and temperature, the 3:1 treatment increased recovered solid yield by 5.47 percentage points and reduced total lignin content in the recovered solid fraction by 2.58 percentage points. Both differences remained statistically significant after Holm correction (*p* < 0.001). Consequently, this condition represents an attractive shorter-duration alternative to the 6 h treatments, combining approximately 70% solid recovery with a total lignin content in the recovered solid fraction below 2%.

The Pareto analysis (Figure 2) identified only four non-dominated conditions for sugarcane bagasse: 3:1 at 140 °C for 6 h, 3:1 at 160 °C for 4 h, 3:1 at 160 °C for 6 h, and 2:1 at 160 °C for 6 h. Unlike the broad Pareto front observed for Acacia wood, these conditions were concentrated in a relatively narrow region characterized by recovered solid yields of approximately 67–75% and total lignin content in the recovered solid fraction of approximately 1–4%.

This behaviour indicates that sugarcane bagasse allowed relatively high recovered solid yields and extensive delignification to be achieved simultaneously. In contrast, Acacia wood showed a stronger antagonistic relationship between the two responses.

### 3.4. Comparison of the Two Feedstocks and Multiresponse Optimization

After characterizing the response of each biomass independently, the two feedstocks were compared under identical operating conditions. The main experimental conditions selected according to maximum recovered solid yield, minimum total lignin content, and maximum equal-weight desirability, are summarized in Table 2. The selected conditions differed markedly between the two feedstocks, confirming that the preferred treatment depends not only on the optimization criterion but also on the feedstock, which is likely related to the different structural characteristics of the two biomasses. In Acacia wood, the conditions maximizing solid recovery and minimizing lignin were located at opposite regions of the experimental domain. On the other hand, in sugarcane bagasse, relatively high solid recovery and low lignin contents could be achieved simultaneously under the most favourable conditions.

For Acacia wood, the highest recovered solid yield and the lowest total lignin content were obtained under markedly different conditions, illustrating the strong trade-off between both responses. The maximum equal-weight desirability therefore represented an intermediate compromise rather than the maximum of either individual response. For sugarcane bagasse, the condition providing the highest recovered solid yield also presented the highest equal-weight desirability, indicating that solid recovery and lignin reduction were less antagonistic than in Acacia wood. Importantly, the combined LA:ChCl treatment and alkaline washing reduced the total lignin content of the recovered solids to below 1% for Acacia wood and below 1.5% for sugarcane bagasse, demonstrating that the combined DES treatment and alkaline washing sequence was effective in reducing the residual lignin content of both feedstocks investigated. However, the solid recovery at which these low lignin contents were reached differed markedly (approximately 30% for Acacia wood and 67% for sugarcane bagasse), and the significant feedstock-dependent interactions show that the ratio, time, and temperature combination required for the best performance must be established separately for each feedstock.

Although a complete component mass balance could not be established, the fraction of the initial lignin removed from the solid can be estimated as LR (%) = 100 − (Y × LS)/L0, where Y is the recovered solid yield (%), LS is the total lignin content of the recovered solid (%), and L0 is the lignin content of the raw material (%). For Acacia wood, LR ranged from approximately 35% under the condition of maximum solid recovery to 99% under the condition of minimum lignin content, with 83% under the condition of maximum desirability. For sugarcane bagasse, LR reached approximately 89%, 96%, and 95% for the 2:1/160 °C/6 h, 3:1/140 °C/6 h, and 3:1/160 °C/4 h conditions, respectively. These values indicate that most of the lignin was removed from the solid under the favourable conditions, but they reflect the combined effect of the DES treatment and the alkaline washing. The unexplained increase in recovered solid yield with treatment severity introduces an additional limitation, as the apparent advantages of the selected conditions may partly reflect changes occurring during post-treatment rather than improved fractionation selectivity.

Conversely, cellulose and hemicellulose recoveries could not be calculated because the carbohydrate composition of the recovered solids was not determined, and the lignin recovered in the liquid fraction was not quantified. Therefore, the loss of solid mass cannot be apportioned between lignin removal, carbohydrate dissolution or degradation, removal of extractives and ash, and losses during washing, and the fractionation selectivity discussed in this work should be regarded as apparent.

Quadratic response-surface models were also evaluated to determine whether the experimental domain could be described through continuous response functions. However, significant lack of fit was observed for seven of the eight combinations of feedstock, solvent ratio, and response. Only the model describing total lignin content in sugarcane bagasse treated with the 3:1 ratio showed no significant lack of fit. Consequently, the quadratic models were not considered sufficiently reliable for continuous prediction or extrapolation of optimum conditions.

The favourable operating regions identified in this study were based on the experimentally evaluated conditions, as represented by the heat maps and Pareto analysis. Therefore, the selected conditions should be regarded as the best-performing combinations among those experimentally evaluated, according to the response criteria considered, rather than as independently validated process optima. Although the replicated factorial design allowed the estimation of experimental variability and the statistical assessment of the investigated factors, additional confirmation experiments would be necessary to verify the reproducibility of the selected conditions and assess their suitability for further process development.

### 3.5. Recovered Fractions and Considerations for Process Implementation

The present study was designed to assess the robustness of the LA:ChCl solvent system across two structurally distinct feedstocks over a broad experimental domain (60 operating conditions, each performed in duplicate). For this reason, process performance was evaluated through recovered solid yield and residual total lignin content, which are the two responses that directly describe the trade-off between solid recovery and delignification. It should be acknowledged, however, that these responses do not describe the carbohydrate composition, crystallinity, or degree of polymerization of the recovered solid fractions, nor the purity and chemical structure of the extracted lignin, which were not determined in this screening study.

Information on these properties is nevertheless available from our previous studies on Acacia wood using the same solvent system. After fractionation with LA:ChCl at a 3:1 molar ratio (160 °C, 4 h, albeit at a higher biomass loading), the cellulose-rich fraction contained approximately 96% cellulose, 2.6% hemicellulose, and 1.4% lignin, corresponding to a delignification of approximately 96% [19]. X-ray diffraction showed that the cellulose I structure was preserved with the 2:1 ratio, whereas the 3:1 ratio led to the appearance of cellulose II features and a lower degree of polymerization [21]. This partial structural modification may also contribute to the lower recovered solid yields observed here for Acacia wood with the 3:1 ratio, and indicates that, beyond the residual lignin content, the solvent ratio may affect the suitability of the cellulose-rich fraction for specific applications. For sugarcane bagasse, the cellulose-rich fraction obtained with LA:ChCl 2:1 (160 °C, 4 h) followed by the same washing sequence was successfully used as the starting material for the production of anionic bio-flocculants [22], demonstrating its suitability for subsequent chemical modification.

Regarding the lignin-rich fraction, lignins recovered from Acacia wood by water-induced precipitation after LA:ChCl treatment (2:1 and 3:1) were previously characterized by 31P NMR, 2D HSQC NMR, and intrinsic viscosity measurements [19]. These lignins presented a higher content of phenolic hydroxyl groups and a lower content of aliphatic hydroxyl groups than a commercial kraft lignin, with estimated molecular weights of approximately 9.4–9.6 kDa [19], features that are of interest for applications such as resins, adhesives, and antioxidant additives. In addition, the purity of the lignin-rich fraction increased slightly during successive solvent reuse cycles [21]. Nonetheless, the purity (e.g., residual carbohydrate and ash contents) and structure (e.g., S/G/H composition and molar mass distribution) of the lignins extracted from sugarcane bagasse, as well as the composition and crystallinity of the recovered solid fractions obtained under the favourable conditions identified here for both feedstocks, remain to be determined. Since grass lignins typically contain *p*-hydroxyphenyl units and hydroxycinnamates that are scarce in hardwood lignins, feedstock-dependent differences in lignin structure are expected and will be addressed in future work.

From an industrial perspective, several bottlenecks must be addressed before the LA:ChCl system can be implemented at scale. The first concerns solvent recovery and reuse. In the procedure used here, the DES is diluted with water during washing and lignin precipitation, so its reuse requires the removal of large amounts of water, typically by evaporation, which is energy-intensive [28]. In our previous study, LA:ChCl (2:1) was reused for five consecutive cycles without purification while maintaining a stable LA concentration; however, glucose, xylose, 5-hydroxymethylfurfural, and acetic acid accumulated in the solvent, and the purity of the cellulose-rich fraction decreased by approximately 10% [21]. Purge streams or intermediate purification steps (e.g., antisolvent precipitation, membrane separation, or adsorption) will therefore be needed to control the build-up of solubilized carbohydrates and degradation products [28]. The carbohydrates dissolved in the DES, particularly under the most severe conditions applied to Acacia wood, where solid recovery decreased to approximately 30%, also represent a carbon fraction that is currently not valorized, highlighting the need for complete mass balances.

A second set of bottlenecks relates to process intensity. The biomass loading used in this work (0.75 g in 10 mL of DES) corresponds to a high solvent-to-biomass ratio, and increasing the solids loading is limited by the relatively high viscosity of LA:ChCl (approximately 88–153 mPa·s at 20 °C, depending on the molar ratio [19]), which hinders mixing, heat transfer, and mass transfer [24,25]. The most favourable conditions identified here also require 140–160 °C for 4–6 h, implying a significant thermal energy demand; in this respect, the 3:1, 160 °C, 4 h condition identified for sugarcane bagasse is attractive because it shortens the treatment without compromising performance. Furthermore, the post-treatment sequence, involving water washing, an alkaline (10 wt% NaOH) wash, and 5–6 additional water washes, consumes chemicals and generates considerable volumes of aqueous effluent, and should be minimized or integrated with solvent and lignin recovery. Finally, the cost of LA, which is higher than that of conventional mineral acids, makes solvent losses and recovery efficiency key economic drivers [24,28]. Addressing these aspects through multi-cycle recycling experiments with both feedstocks, process intensification at higher solids loadings, and techno-economic and life-cycle assessments will be essential to translate the feedstock flexibility demonstrated here into a viable multi-feedstock biorefinery.

## 4. Conclusions

The fractionation of two structurally distinct lignocellulosic feedstocks, Acacia wood and sugarcane bagasse, was investigated using LA:ChCl-based DESs at molar ratios of 2:1 and 3:1, followed by alkaline washing, under different extraction times and temperatures. The combined treatment sequence resulted in substantial lignin removal from both biomasses, reaching residual total lignin contents below 1.5%, although at markedly different recovered solid yields (approximately 30% for Acacia wood and 67% for sugarcane bagasse). Importantly, these results reflect the overall fractionation process, and the individual contribution of the DES treatment to lignin removal could not be determined independently of the subsequent alkaline washing. The statistical analysis showed that the effects of solvent ratio, extraction time, and temperature on the final fractionation performance differed between the two feedstocks, indicating that the most favourable operating conditions are feedstock-dependent.

Acacia wood exhibited a clear trade-off between recovered solid yield and total lignin content, whereas sugarcane bagasse allowed relatively high solid recovery and low lignin contents to be achieved simultaneously. The highest equal-weight desirability was obtained at 3:1, 120 °C, and 6 h for Acacia wood, and at 2:1, 160 °C, and 6 h for sugarcane bagasse. These conditions represent the most favourable experimental combinations identified within the investigated domain and should not be interpreted as independently validated process optima. Additional confirmation experiments are required to establish their reproducibility and suitability for further process development.

Overall, the results show that LA:ChCl DESs were effective for both a hardwood (Acacia) and an agricultural residue (sugarcane bagasse), two feedstocks with markedly different composition, provided that the operating conditions are optimized separately for each feedstock and according to the relative importance assigned to solid recovery and lignin removal. The residual lignin contents reported here reflect the combined DES treatment and alkaline washing, and whether this applicability extends to other lignocellulosic feedstocks, and whether the process can be operated sustainably at larger scale, remain hypotheses that require further investigation, including control experiments to quantify the separate contributions of the DES treatment and the alkaline washing. Further studies should validate the selected conditions and evaluate solvent recovery and reuse over multiple cycles for both feedstocks, complete mass and energy balances, the composition, structure, and purity of the recovered solid and lignin-rich fractions, and the techno-economic and environmental performance of the process.

## Figures and Tables

**Table 1:** Chemical composition of Acacia wood and sugarcane bagasse [19,22].

Biomass	Composition (wt%)		
	Lignin	Cellulose	Hemicellulose
Acacia wood	22.20	47.41	18.79
Sugarcane bagasse	24.02	38.78	23.81

**Table 2:** Selected experimental conditions corresponding to the maximum recovered solid yield, minimum total lignin content in the recovered solid fraction, and maximum equal-weight desirability. Values are expressed as mean ± standard deviation of two independent extractions.

Feedstock	Selection Criterion	LA:ChCl	Time (h)	Temperature (°C)	Recovered Solid Yield (%)	Total Lignin (%)
Acacia wood	Maximum recovered solid yield	2:1	2	120	89.20 ± 0.07	16.26 ± 0.20
	Minimum total lignin content	3:1	8	140	30.05 ± 1.65	0.94 ± 0.21
	Maximum equal-weight desirability	3:1	6	120	52.93 ± 1.70	7.14 ± 0.36
Sugarcane bagasse	Maximum recovered solid yield	2:1	6	160	75.20 ± 3.07	3.64 ± 0.65
	Minimum total lignin content	3:1	6	140	67.48 ± 0.15	1.32 ± 0.77
	Maximum equal-weight desirability	2:1	6	160	75.20 ± 3.07	3.64 ± 0.65

## Data Availability

The original contributions presented in this study are included in the article. Further inquiries can be directed to the corresponding authors.

## Supplementary Materials

The following supporting information can be downloaded at: https://www.mdpi.com/article/10.3390/polym18212729/s1, Table S1: Complete experimental results for Acacia wood and sugarcane bagasse fractionation.
