# Supplementary material for: Fractionation of Acacia Wood and Sugarcane Bagasse by Levulinic Acid–Choline Chloride Deep Eutectic Solvent Treatment Followed by Alkaline Washing
Source: Polymers (Basel). 2026 Nov 3;18(21):2729. doi: 10.3390/polym18212729 (PMC13765703; doi:10.3390/polym18212729)
Supplement: Supplementary file 1 [file polymers-18-02729-s001.zip › polymers-4618665-supplementary.pdf]

Table S1. Complete experimental results for Acacia wood and sugarcane bagasse fractionation.

Feedstock	LevA:ChCl ratio	Time (h)	Temperature (°C)	Recovered solid yield (%)	Total lignin content in the cellulose-rich fraction (%)
Acacia wood	2:1	1	120	88.50 ± 0.24	17.35 ± 0.34
	2:1	1	140	82.11 ± 1.06	15.87 ± 0.37
	2:1	1	160	53.44 ± 0.03	9.04 ± 0.06
	2:1	2	120	89.20 ± 0.07	16.26 ± 0.20
	2:1	2	140	54.71 ± 0.51	14.95 ± 1.83
	2:1	2	160	49.70 ± 4.36	7.61 ± 1.28
	2:1	4	120	84.27 ± 1.58	15.89 ± 0.79
	2:1	4	140	45.20 ± 0.51	9.62 ± 0.30
	2:1	4	160	40.35 ± 0.34	7.41 ± 0.88
	2:1	6	120	68.86 ± 0.13	15.30 ± 0.21
	2:1	6	140	38.14 ± 0.26	8.34 ± 0.51
	2:1	6	160	23.49 ± 0.70	6.01 ± 0.64
	2:1	8	120	53.79 ± 1.69	15.09 ± 0.15
	2:1	8	140	44.36 ± 1.44	6.53 ± 0.55
	2:1	8	160	27.27 ± 0.26	5.82 ± 0.73
	3:1	1	120	74.82 ± 3.46	20.56 ± 0.49
	3:1	1	140	58.56 ± 0.67	18.79 ± 0.47
	3:1	1	160	34.88 ± 1.04	6.78 ± 0.23
	3:1	2	120	68.69 ± 1.71	15.78 ± 0.37
	3:1	2	140	52.02 ± 1.83	9.01 ± 0.94
	3:1	2	160	32.99 ± 0.31	4.00 ± 0.02
	3:1	4	120	64.52 ± 0.03	12.34 ± 0.49
	3:1	4	140	32.93 ± 2.67	5.97 ± 0.44
	3:1	4	160	31.16 ± 0.06	2.35 ± 0.72
	3:1	6	120	52.93 ± 1.70	7.14 ± 0.36
	3:1	6	140	30.32 ± 1.06	1.33 ± 0.13
	3:1	6	160	20.16 ± 1.08	1.03 ± 0.02
	3:1	8	120	37.91 ± 1.95	5.97 ± 0.21
	3:1	8	140	30.05 ± 1.65	0.94 ± 0.21
	3:1	8	160	17.72 ± 1.26	1.13 ± 0.02
Sugarcane bagasse	2:1	1	120	28.66 ± 0.14	21.86 ± 0.14
	2:1	1	140	52.62 ± 7.09	12.16 ± 2.08
	2:1	1	160	62.59 ± 1.38	6.04 ± 0.30
	2:1	2	120	29.15 ± 2.80	13.27 ± 0.44
	2:1	2	140	52.93 ± 8.70	5.20 ± 7.36
	2:1	2	160	57.97 ± 2.61	9.06 ± 2.73
	2:1	4	120	42.06 ± 0.24	17.95 ± 1.00
	2:1	4	140	70.24 ± 3.47	5.29 ± 0.01
	2:1	4	160	64.11 ± 0.08	4.39 ± 0.11
	2:1	6	120	36.13 ± 4.32	10.61 ± 4.13
	2:1	6	140	61.78 ± 4.10	2.68 ± 0.54
	2:1	6	160	75.20 ± 3.07	3.64 ± 0.65
	2:1	8	120	52.38 ± 1.07	3.82 ± 2.30
	2:1	8	140	63.05 ± 3.74	6.50 ± 0.76
	2:1	8	160	65.22 ± 2.43	4.63 ± 1.39
	3:1	1	120	29.69 ± 4.59	18.89 ± 1.02
	3:1	1	140	66.06 ± 2.09	11.87 ± 2.79
	3:1	1	160	65.64 ± 1.04	5.55 ± 0.40
	3:1	2	120	43.08 ± 3.73	12.49 ± 1.22
	3:1	2	140	62.76 ± 3.44	6.92 ± 0.49
	3:1	2	160	66.85 ± 0.81	4.72 ± 0.94
	3:1	4	120	53.14 ± 1.51	11.85 ± 0.54
	3:1	4	140	62.42 ± 0.54	2.96 ± 0.96

3:1	4	160	69.58 ± 0.11	1.81 ± 0.12
3:1	6	120	55.19 ± 0.04	8.71 ± 0.11
3:1	6	140	67.48 ± 0.15	1.32 ± 0.77
3:1	6	160	70.21 ± 0.27	2.72 ± 0.68
3:1	8	120	62.05 ± 0.73	9.37 ± 2.77
3:1	8	140	63.84 ± 3.71	3.93 ± 2.49
3:1	8	160	72.00 ± 6.42	6.01 ± 1.20
